# Development of an AI-driven digital assistance system for real-time safety evaluation and quality control in laparoscopic liver surgery

**DOI:** 10.3389/fonc.2025.1678525

**Published:** 2025-10-08

**Authors:** Zi-Yang Peng, Zhi-Bo Wang, Yan Yan, Hao-Qian Peng, Yong-Tai Ma, Yu-Tong Li, Yao-Xing Ren, Jun-Xi Xiang, Kun Guo, Gang Wang, Jian-Feng Duan, Xiao-Wen Li, Yu Guan, Xue-Min Liu, Rong-Qian Wu, Yi Lyu, Li Yu

**Affiliations:** ^1^ Department of Otorhinolaryngology-Head and Neck Surgery, The First Affiliated Hospital of Xi’an Jiaotong University, Xi’an, Shaanxi, China; ^2^ School of Future Technology, National Local Joint Engineering Research Center for Precision Surgery & Regenerative Medicine, Xi’an Jiaotong University, Xi’an, Shaanxi, China; ^3^ Health Science Center, Xi’an Jiaotong University, Xi’an, Shaanxi, China; ^4^ Department of Hepatobiliary Surgery, The First Affiliated Hospital of Xi’an Jiaotong University, Xi’an, Shaanxi, China; ^5^ Department of Hepatobiliary and Pancreatic Surgery II, Baoji Central Hospital, Baoji, Shaanxi, China; ^6^ Department of Hepatobiliary Surgery, Hanzhong 3201 Hospital, Hanzhong, Shaanxi, China; ^7^ Department of Geriatric General Surgery, The Second Affiliated Hospital of Xi’an Jiaotong University, Xi’an, Shaanxi, China; ^8^ Department of Anesthesiology and Perioperative Medicine, The First Affiliated Hospital of Xi’an Jiaotong University, Xi’an, Shaanxi, China

**Keywords:** digital surgery, AI assistance, intraoperative quality control, surgical decision support, real-time safety evaluation

## Abstract

**Background:**

By performing AI-driven workflow analysis, intelligent surgical systems can provide real-time intraoperative quality control and alerts. We have upgraded an Intelligent Surgical Assistant (ISA) through integrating a redesigned hierarchical recognition algorithm, an expanded surgical dataset, and an optimized real-time intraoperative feedback framework.

**Objective:**

We aimed to assess the accuracy of the ISA in real-time instrument tracking, organ segmentation, and phase classification during laparoscopic hemi-hepatectomy.

**Methods:**

In this retrospective multi-center analysis, a total of 142861 annotated frames were collected from 403 laparoscopic hemi-hepatectomy videos across 4 centers to build a comprehensive database of surgical video annotations. Each frame was labeled for surgical phase, organs, and instruments. The algorithm in the ISA was retrained using a hybrid deep learning framework integrating instrument tracking, organ segmentation, and phase classification. We then established a scoring system for surgical image recognition and evaluated the algorithm’s recognition accuracy and inter-operator consistency across different surgical teams.

**Results:**

The upgraded ISA achieved an accuracy of 89% in real-time recognition of instruments and organs. The programmatic phase classification for laparoscopic hemi-hepatectomy reached an average accuracy of 91% (p<0.001), enabling a correct recognition of surgical events. The inter-operator variability in recognition was reduced to 14.3%, highlighting the potential of AI-assisted quality control to standardize intraoperative alerts. Overall, the ISA demonstrated high precision and consistency in phase recognition and operative field evaluation across all phases (accuracy >87%, specificity ~90% in each phase). Notably, critical phases (Phase 1 and Phase 5) were identified with an exceptional accuracy area under the curve (AUC 0.96 in Phase 1; AUC 0.87 in Phase 5), indicating that key surgical procedures could be phased with very low false-alarm rates.

**Conclusions:**

The optimized ISA provides a highly accurate real-time interpretation of surgical phases and a strong potential to standardize surgical procedures, thus guaranteeing the outcomes and safety of laparoscopic hemi-hepatectomy.

## 1 Introduction

Since the first laparoscopic cholecystectomy accomplished by Philippe Mouret in 1987, minimally invasive techniques have flourished, allowing an array of surgical procedures from simple elective to complex comprehensive, such as tumor and organ removal (1–3). Surgical robots have further polished these procedures. The da Vinci system, FDA-approved in 2000, offers 3D high-definition vision, wristed instruments, and tremor filtering, and can markedly increase the precision of surgical procedures (4). Clinical studies have confirmed that, compared with open surgery, minimally invasive and robotic surgery boasts a lower perioperative complication rate (from 15.2% down to 9.8%), a shorter hospital stay (on average by 2.3 days), and a milder postoperative pain (VAS reduced by 1.7 points) (5). These advantages have been translated into a lower morbidity and a faster recovery (6, 7). Meta-analyses show that laparoscopic approaches can be safely applied in liver surgery, even among patients with malignant diseases, offering similar oncological outcomes with less blood loss and shorter hospitalization, compared to open surgery (8, 9).

However, traditional laparoscopy is still challenged by a “fulcrum effect” (opposite motion beneath trocar fulcrums) (10), limited tactile feedback, and a two-dimensional operative view. It is particularly difficulty to overcome these challenges when dissecting dense adhesions or structures with complex anatomies (11–14). Cognitive errors and fatigue of surgeons may affect the surgical outcomes (15). Therefore, artificial intelligence systems may be integrated with laparoscopy to bring more surgical benefits (16).

While AI models have demonstrated significant success in discrete tasks such as tool tracking or anatomical segmentation, a key challenge remains: integrating these functions into a single, cohesive system that maintains both high accuracy across multiple tasks and the real-time inference speed required for clinical utility. Many existing systems excel at one task but often struggle to perform comprehensive, multi-faceted analysis without sacrificing speed (17, 18). To address this gap, we developed and validated an Intelligent Surgical Assistant (ISA) for laparoscopic hemi-hepatectomy. Our system is specifically designed to perform simultaneous instrument tracking, organ segmentation, and surgical phase classification, all while operating at a clinically viable frame rate. By providing this holistic, real-time analysis, the ISA aims to deliver timely and relevant feedback to surgeons, enhancing intraoperative quality control and safety (19).

This ISA has been trained to distinguish six stages on the phase-labeled videos of laparoscopic hemi-hepatectomy: Phase 1 (intraoperative ultrasound), Phase 2 (first hepatic hilum dissection), Phase 3 (second hilum dissection), Phase 4 (exposure of the middle hepatic vein), Phase 5 (post-resection hemostasis on liver cut surface), and Phase 0 (non-critical steps). In clinical settings, ISA processes the incoming laparoscopic video frame-by-frame at ≥30 FPS, meanwhile labeling the current phase and offering a clarity score in real time. The surgeon can thus verify if a critical phase has been satisfactorily completed. For example, a high clarity score in “Phase 5” indicates a clear surgical field, in which liver transection is complete and hemostasis is successful. Conversely, a low clarity score indicates the presence of smoke or bleeding, warning that the surgeon should pause to clear them. Using ISA, a surgeon can recognize the phase and check the field more precisely, thereby ensuring the safety of all procedures.

## 2 Methods

### 2.1 Study design and ethics

This observational study involved no additional interventions beyond standard laparoscopic hemi-hepatectomy. All patient data were de-identified before analysis in compliance with local data privacy regulations and the Declaration of Helsinki. Data use was approved by the Clinical Research Ethics Committee of Xi’an Jiaotong University, Approval Date: July 15, 2023, Approval No. XJTU1AF2023LSK-429. All patients (aged >18 years) had shown consent to our videoing surgical procedures for research purposes. All participating surgeons provided informed consent for the retrospective use of their surgical videos in workflow evaluation and frame recognition. Video data were retrospectively collected between Aug 30, 2023, and Aug 7, 2024.

### 2.2 Dataset construction (laparoscopic hemi-hepatectomy)

This retrospective study included a cohort of 403 patients who underwent laparoscopic hemi-hepatectomy between August 2023 and August 2024, from which 403 surgical videos were obtained from four participating centers. The inclusion criteria were: (1) adult patients (age > 18 years); (2) undergoing elective laparoscopic hemi-hepatectomy; (3) availability of complete, high-quality surgical video recordings; and (4) provision of informed consent for the research use of video data. Exclusion criteria were: (1) emergency surgeries; (2) procedures converted to open surgery due to non-oncological reasons (e.g., equipment failure); (3) patients with prior major upper abdominal surgery; and (4) videos with significant portions obscured by technical issues or poor quality. The baseline demographic and clinical characteristics of the patient cohort are summarized in **Table 1**.

**Table 1:** Baseline characteristics of the patient cohort (N = 403).

Characteristic	Value
Age, years (Mean ± SD)	58.4 ± 11.2
Sex (Male), n (%)	282 (70.0%)
BMI, kg/m² (Mean ± SD)	24.1 ± 3.5
Hepatocellular Carcinoma, n (%)	250 (62.0%)
Benign Lesion, n (%)	40 (10.0%)

Summary of baseline demographic and clinical characteristics for the 403 patients included in the study.

Data are presented as mean ± standard deviation (SD) for continuous variables, and as count (n) and percentage (%) for categorical variables.

From these videos, the internal deep learning cohort was constructed through a rigorous, two-step quality control process. First, an initial frame selection was conducted by junior surgeons (PZY, YY, PHQ, MYT, LYT). They were tasked with selecting representative frames for each key surgical phase, based on predefined visual criteria designed to ensure anatomical clarity, as detailed in our scoring system (now **Table 2**). For instance, frames selected for Phase 2 (‘First hepatic hilum dissection’) were required to show a clear exposure of the hilar structures. Each frame selected in this initial step also had to meet a confidence level exceeding 50% for clear identification.

**Table 2:** Scoring criteria for surgical phases.

Score	Description
Phase 0:	Non-operative (non-key) phase
2	Clearly identified as a non-critical stage frame, but still of reference value
1	Some anatomical structure is visible, but none corresponds to any key surgical phase
0	No recognizable anatomical structure is visible in the frame; the view is severely occluded
Phase 1:	Intraoperative ultrasound inspection
2	The ultrasound probe is in good contact with the liver surface, the target area under examination is clearly displayed, and the examination process is fully visible.
1	The ultrasound probe can be seen on the liver surface, but its contact area or angle is suboptimal, and the key area of examination is not fully displayed.
0	The ultrasound probe or the area under examination cannot be identified in the laparoscopic view.
Phase 2:	First hepatic hilum dissection
2	The anatomical structures of the first hepatic hilum region are clearly exposed, and the occlusion band is intact and clearly visible.
1	Some anatomical structures of the first hepatic hilum or the occlusion band can be seen, but the view is incomplete or unclear.
0	Key structures of the first hepatic hilum are not sufficiently exposed, and the occlusion band cannot be identified.
Phase 3:	Second hepatic hilum dissection
2	The second hepatic hilum region is fully mobilized, and the major vascular structures (the inferior vena cava and the hepatic vein confluence) are clearly visible with well-defined boundaries.
1	Some anatomical structures in the second hepatic hilum region are exposed, but key structures are only partially visible or unclear.
0	Major structures in the second hepatic hilum region are not exposed and cannot be identified.
Phase 4:	Middle hepatic vein exposure
2	The middle hepatic vein is fully dissected and clearly exposed, and its entire course is clearly visible.
1	The approximate location of the middle hepatic vein can be discerned, but it is not completely or clearly displayed.
0	The middle hepatic vein is not seen in the surgical field, and its anatomy is not exposed.
Phase 5:	Electrocautery on transection surface after isolated diseased liver
2	The entire liver transection surface is unobstructed and clearly displayed, and all points requiring electrocautery hemostasis are clearly visible and have been treated.
1	Part of the liver transection surface is clearly shown, but some local areas are still obstructed or blurred, and details of the electrocautery treatment are not fully visible.
0	After resection, the liver transection surface is not clearly displayed, and the electrocautery hemostasis sites are difficult to identify

For each phase, frames are categorized by visual clarity: Score 2 implies that the relevant anatomy or task is clearly exposed (e.g., in Phase 1 the ultrasound probe fully contacts the liver surface). Score 1 indicates incomplete or unclear exposure of key elements, and Score 0 indicates no recognizable structures.

These initially selected frames then underwent a second-step review and supervision by senior surgeons (XJX, GK, LXM, LY). The senior surgeons’ review protocol was twofold. First, they qualitatively validated that each frame was a high-quality, representative example of its designated phase, rejecting images with visual obstructions such as excessive smoke, blood, or off-target camera angles. Second, they applied a much stricter quantitative threshold, excluding any frame with a final confidence level below 90%. During this stage, images exhibiting similar surgical features were also reduced to minimize redundancy. This stringent quality control process resulted in the exclusion of 5,934 frames, with the final 136,927 high-confidence frames being retained for annotation.

**Figure 1:**
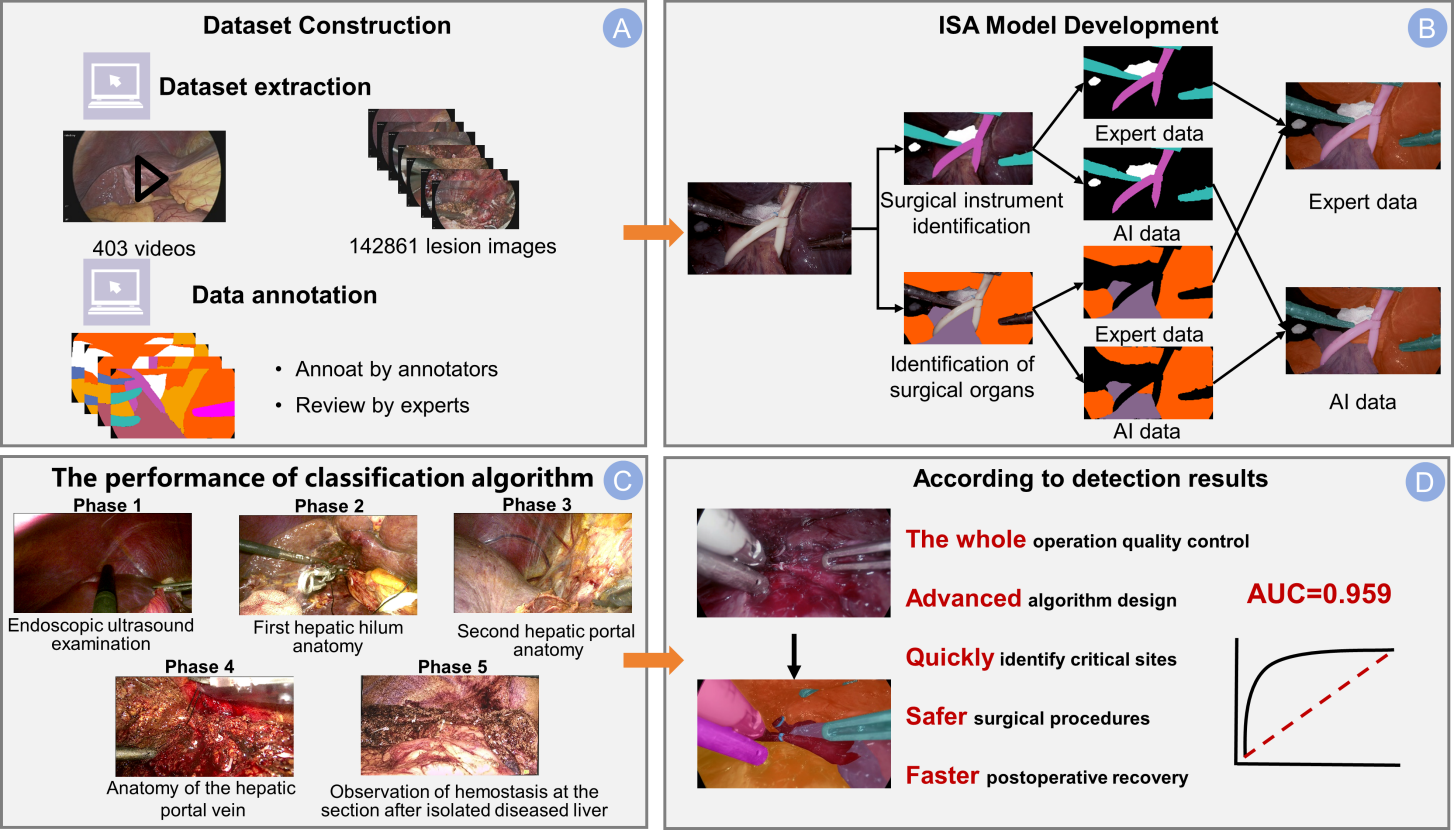
Integrated dataset and ISA model overview. **(A)** The dataset construction pipeline. The dataset was sourced from 403 surgical videos, and the process included frame extraction, annotation by junior surgeons, and final review by experts. **(B)** The development framework of the Intelligent Surgical Assistant (ISA) model with separate branches for identifying surgical instruments and anatomical structures. Example outputs are shown as expert-annotated masks (Expert data), compared to the model’s predicted segmentation (AI data) for both tasks. **(C)** The classification performance across five surgical phases: Phase 1 (Endoscopic ultrasound examination), Phase 2 (First hepatic hilum anatomy), Phase 3 (Second hepatic portal anatomy), Phase 4 (Anatomy of the hepatic portal vein), and Phase 5 (Observation of hemostasis at the resection site after removal of the diseased liver). **(D)** The significance of the detection results: this advanced algorithmic system enables whole-process quality control, accurately identifies critical sites (arrow), and contributes to safer surgical procedures and faster postoperative recovery. The model achieves an AUC of 0.959, demonstrating a robust discriminatory capability.

Each of the retained frames was annotated with segmentation masks in distinct colors using LabelMe software, highlighting critical areas such as liver parenchyma (primary target), biliary structures, major blood vessels, surgical instruments, and background structures (**Figure 1B**). The inter-operator consistency of the review process was assessed using Fleiss’ Kappa. To quantify this, we calculated the coefficient on a randomly selected subset of 10% of the annotated frames, yielding a Kappa value of 0.88 (p < 0.001), indicating almost perfect agreement.

**Figure 2:**
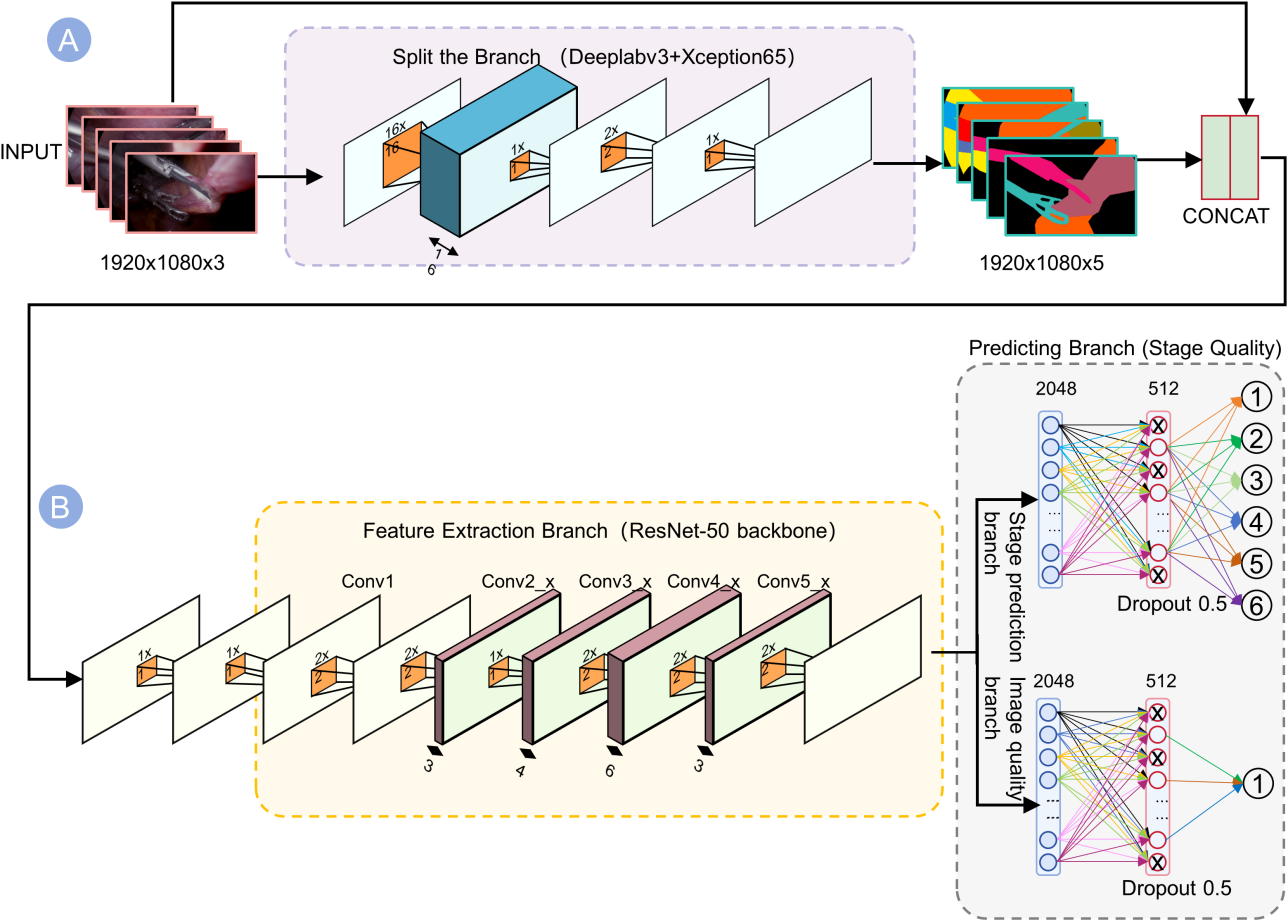
Dual-branch network architecture for joint prediction of stages and quality. **(A)** The top branch (Segmentation) uses DeepLabv3+ with Xception65 backbone to generate 5-channel outputs highlighting tools and anatomy. **(B)** The bottom branch (Feature Extraction) passes the image through ResNet-50 convolutional layers (Conv1–Conv5) to capture contextual information. These outputs are concatenated into a 1920×1080×5 tensor, which is then processed by two parallel dense “Predicting Branches” ending in softmax layers. By fusing segmentation cues with deep semantic features, the network robustly predicts the visual quality of each surgical phase.

Each annotated frame was then labeled according to the surgical phase, with the dataset stratified to ensure representation of the five primary surgical stages (Phases 1-5). To enhance the model’s generalizability, the study incorporated 10-fold cross-validation (**Figure 2**). This technique divided the dataset into 10 subsets, with each subset serving as the test set once, while the remaining nine subsets were used for training. This approach facilitated multiple rounds of training and testing, ensuring robust performance across diverse datasets.

### 2.3 Training ISA using AI models

**Figure 3:**
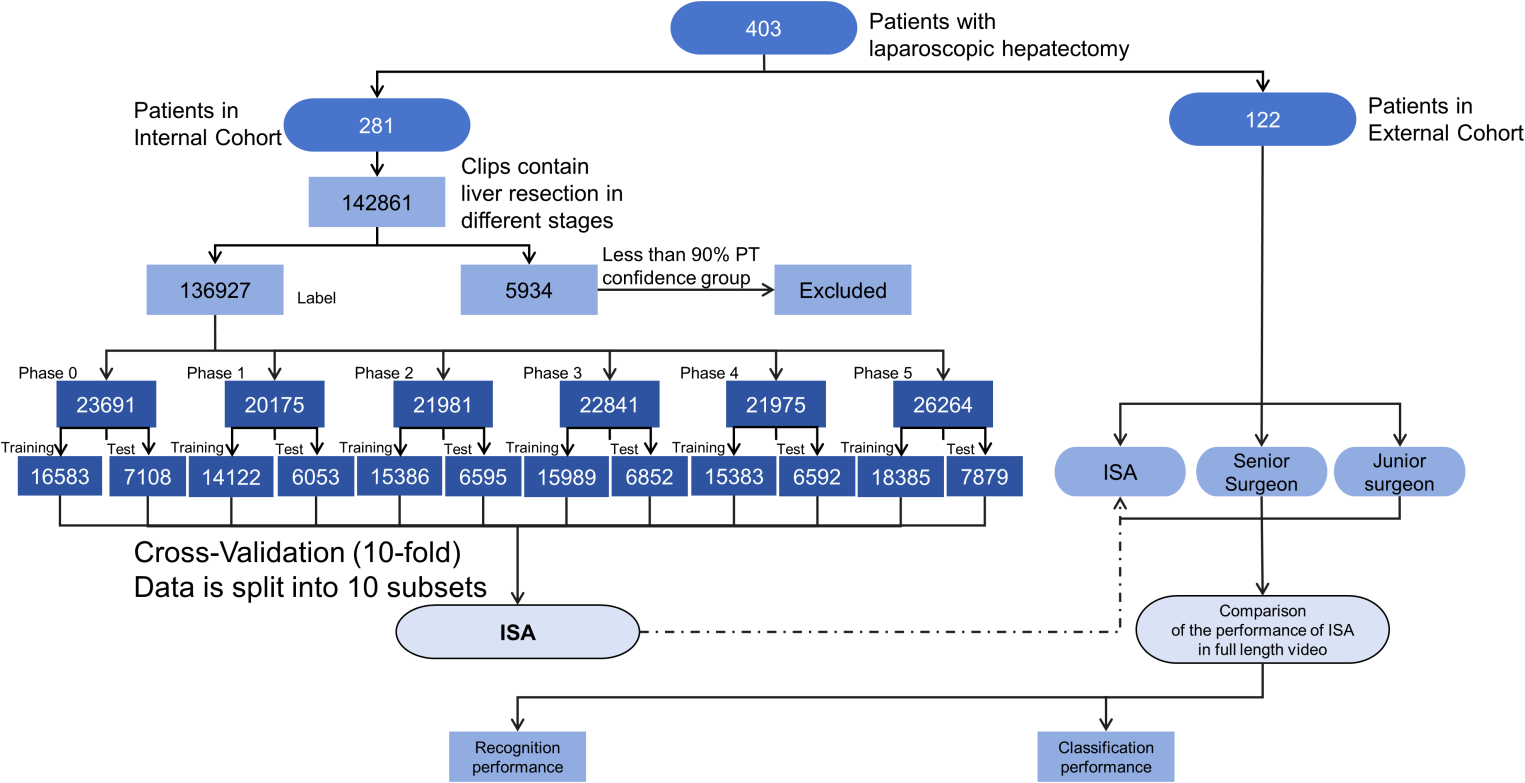
Flowchart of ISA development and evaluation. ISA refers to the Artificial Intelligence model constructed in this study for intraoperative phase recognition during laparoscopic hepatectomy. The flowchart outlines the entire pipeline including patient enrollment, video acquisition, phase-wise annotation, data screening, and model validation. Patients were divided into internal and external cohorts. Only clips with phase transition (PT) confidence above 90% were retained for analysis. A 10-fold cross-validation strategy was applied on the internal dataset for performance evaluation.

We designed a deep learning model of hybrid segmentation and multi-task joint learning (**Figure 3**). Input frames (1920×1080 resolution) were first processed by a “Split-and-Branch” semantic segmentation module to identify and mask the key anatomical structures. The resultant mask was fused with the original image and fed into a pre-trained ResNet-50 backbone to extract deep features (2048-dimensional from the Conv5_x stage). The ResNet’s layers (Conv1 to Conv5_x) were run to extract features in a hierarchical sequence: upper layers to capture low-level edges and textures, while deeper layers to model complex organs and instruments. The segmentation mask emphasizes salient regions, allowing ResNet to selectively catch instrument shapes or liver tissue textures.

The model’s architecture was intentionally designed for computational efficiency to ensure its utility in a real-time clinical setting. This efficiency is primarily achieved through a shared feature extractor and lightweight prediction branches. By using a single ResNet-50 backbone to generate shared features for all downstream tasks, we effectively avoid the redundant computations that would arise from running multiple independent models.

From these shared backbone features, two lightweight fully-connected branches simultaneously (1) determine the surgical phase (Phases 0–5) (**Figure 1C**) and (2) score the image clarity. We trained the model in PyTorch on an NVIDIA Tesla V100 GPU, achieving an average inference latency of approximately 52 ms per frame (corresponding to 19.2 FPS as reported in **Table 3**), which was sufficient for generating intraoperative real-time feedback. The phase classification branch output a probability distribution over the 6 phases, while the quality branch predicted a scalar clarity score reflecting visibility (smoking, bleeding, etc.). We employed joint loss optimization in both tasks, so that the shared features could benefit both phase recognition and clarity assessment.

**Table 3:** Performance of the model.

Algorithm	AP50	AP75	AP50:95	APM	APL	Frame rate
Ours	95.2%*	65.4%*	62.1%*	54.3%	64.8%*	19.2*
SurgeNet (27)	92.8%	61.7%	58.9%	55.6%*	61.2%	17.5
TransUNet (28)	90.5%	58.9%	56.3%	52.8%	57.9%	15.3
EATFormer (29)	88.3%	56.2%	53.7%	50.1%	55.3%	13.8
EndoViT (30)	86.1%	53.8%	51.2%	47.6%	52.7%	11.6
DeepLabv3+ (31)	83.9%	51.5%	48.9%	45.2%	50.4%	10.1

We designed a deep learning model of hybrid segmentation and multi-task joint learning (**Figure 3**). Input frames (1920×1080 resolution) were first processed by a “Split-and-Branch” semantic segmentation module to identify and mask the key anatomical structures. The resultant mask was fused with the original image and fed into a pre-trained ResNet-50 backbone to extract deep features (2048-dimensional from the Conv5_x stage). The ResNet’s layers (Conv1 to Conv5_x) were run to extract features in a hierarchical sequence: upper layers to capture low-level edges and textures, while deeper layers to model complex organs and instruments. The segmentation mask emphasizes salient regions, allowing ResNet to selectively catch instrument shapes or liver tissue textures.

From these shared backbone features, two lightweight fully-connected branches simultaneously (1) determine the surgical phase (Phases 0–5) (**Figure 1C**) and (2) score the image clarity. We trained the model in PyTorch on an NVIDIA Tesla V100 GPU, achieving a millisecond inference (average latency ~52 ms per frame), which was sufficient for generating intraoperative real-time feedback. The phase classification branch output a probability distribution over the 6 phases, while the quality branch predicted a scalar clarity score reflecting visibility (smoking, bleeding, etc.). We employed joint loss optimization in both tasks, so that the shared features could benefit both phase recognition and clarity assessment.

### 2.4 Evaluation metrics

We evaluate model performance using standard object detection metrics. AP50, AP75, and AP50:95 represent the Average Precision (AP) under Intersection-over-Union (IoU) thresholds of 0.5, 0.75, and the average from 0.5 to 0.95 (step = 0.05), respectively. AP is computed as the area under the Precision-Recall (PR) curve:


$$\text{AP}=\int_{0}^{1}\text{p}(\text{r})\text{dr}$$

where p(r)denotes precision at recall r. In addition, APM and APL measure detection accuracy for medium-sized and large-sized objects, respectively, following the COCO evaluation protocol. Frame rate (FPS) reflects inference speed and computational efficiency.

### 2.5 Statistical analysis

The performance of the ISA was evaluated according to accuracy, precision, recall, and F1-score. Phase recognition results were also summarized in a confusion matrix. To ensure performance was superior to random assignment, the statistical significance of the phase classification results was validated using a chi-square test (p < 0.05).

### 2.6 Clarity scoring system for five key intraoperative phases of laparoscopic liver resection

**Table 2** defines a three-point system (0–2) for scoring the clarity in each of the five phases of laparoscopic liver resection, including: Phase 1 (intraoperative liver ultrasound), Phase 2 (first porta hepatis dissection), Phase 3 (second porta hepatis dissection), Phase 4 (middle hepatic vein exposure), and Phase 5 (electrocoagulation of the liver section surface). Phase 0 was set as a non-critical background phase. For each phase, a score of 2 represented a full visualization of anatomical structures and an optimal position of the camera, 1 indicated partial anatomical exposure or a suboptimal position, and 0 denoted indistinguishable anatomical structures or an obstructed vision (e.g., smoke, blood, off-target lens). The clinical validity of this scoring system is rooted in its development by senior hepatobiliary surgeons and its direct correlation with intraoperative safety. The criteria for each score were established through expert consensus based on extensive surgical experience. A high clarity score (Score 2) represents an optimal surgical field, which is a prerequisite for the safe identification of critical anatomical structures and the prevention of iatrogenic injury. Conversely, a low score (0 or 1) signifies a compromised view due to factors like bleeding, smoke, or suboptimal exposure. Such situations are clinically significant as they substantially increase the risk of complications. Therefore, this scoring system serves as a clinically relevant and valid proxy for quantifying the quality and safety of the operative field in real-time.

## 3 Results

### 3.1 Phase classification

**Figure 4:**
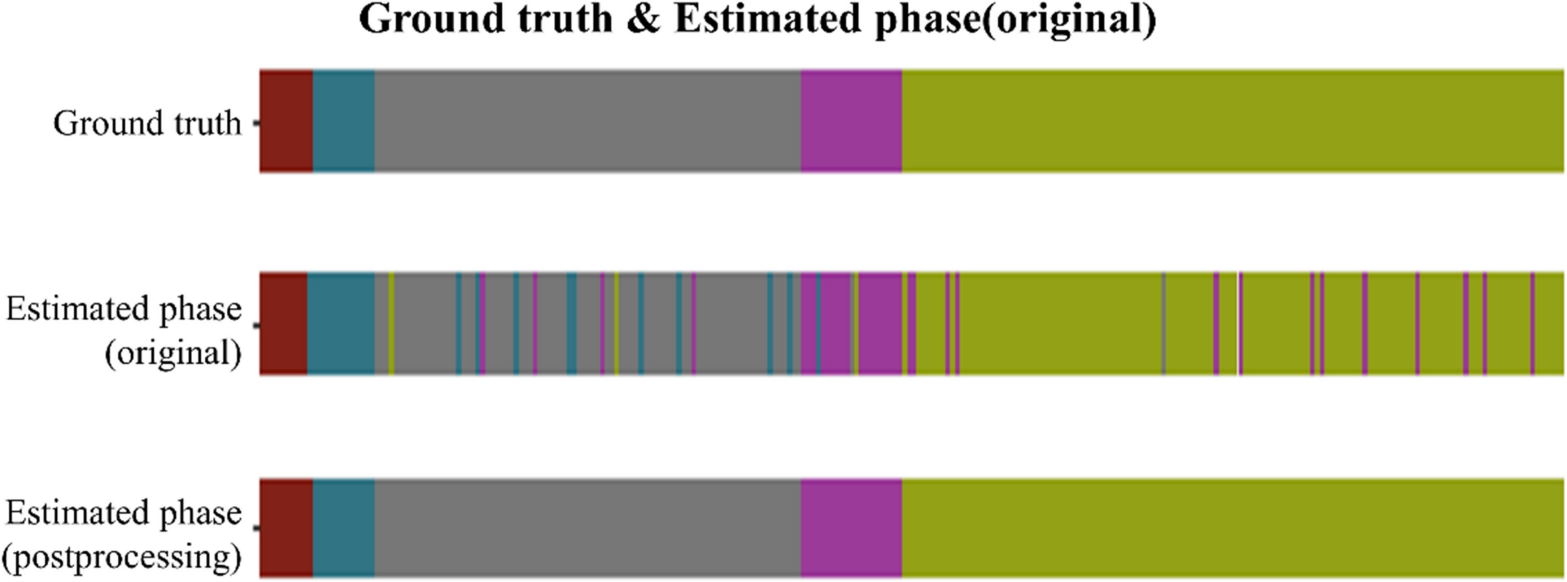
Phase recognition results for one laparoscopic hemi-hepatectomy video. For a representative case from the test set, the three color-coded ribbons illustrate surgical-phase predictions versus ground truth along the temporal axis. The top ribbon shows the ground-truth labels; the middle ribbon presents the primary model predictions; the bottom ribbon displays the refined predictions after post-processing. The laparoscopic cholecystectomy (LC) procedure was temporally divided into five phases: (1) Endoscopic ultrasound examination, (2) First hepatic hilum anatomy, (3) Second hepatic portal anatomy, (4) Anatomy of the hepatic portal vein, and (5) Observation of hemostasis at the section after isolated diseased liver. In the top ribbon of **Figure 5**, these phases are sequentially encoded with five distinct colors.

The ISA demonstrated an average accuracy of 91% in classifying the five key phases (p<0.001). **Figure 4** presents phase recognition results for a representative test case, with color-coded ribbons comparing the model’s predictions to the ground-truth annotations. Performance details are shown in **Table 4** (confusion matrix). The ISA matched most of the frames with the phase correctly, with an accuracy of 89.0% in Phase 1 and 90.5% in Phase 5, indicating its high reliability in phase segmentation. Misclassifications were rare (<8% for any off-diagonal), primarily in frames at the transition between two phases. Overall, the model could clearly distinguish between major procedural steps of laparoscopic hemi-hepatectomy, with a recall >82% in each phase.

**Table 4:** Confusion matrix of phase prediction (percent).

Accurate phase \ Prediction phase	Phase 0	Phase 1	Phase 2	Phase 3	Phase 4	Phase 5
Phase 0	96.1%	1.0%	0.6%	0.6%	0.8%	0.9%
Phase 1	6.5%	89.0%	2.0%	1.0%	1.0%	0.5%
Phase 2	8.2%	1.5%	87.5%	1.5%	0.8%	0.5%
Phase 3	11.4%	0.8%	1.8%	83.5%	1.5%	1.0%
Phase 4	7.2%	0.5%	0.6%	1.2%	88.0%	2.5%
Phase 5	6.0%	0.5%	0.5%	0.5%	2.0%	90.5%

The confusion matrix highlights accurate phase identification. High values along the diagonal (e.g., 96.1% for Phase 0, 89.0% for Phase 1, 88.0% for Phase 4, 90.5% for Phase 5) indicate most frames are correctly classified into their true phase. Off-diagonal percentages are very low (<10%), showing few misclassifications between phases.

While overall misclassifications were rare (<8% for any off-diagonal), a closer analysis of the confusion matrix (**Table 4**) reveals that the most frequent misclassification occurred between Phase 3 and Phase 0 (11.4%), suggesting that the final moments of the second hilum dissection can be visually similar to non-critical operative steps. Similarly, some confusion was observed between Phase 2 and Phase 0 (8.2%). These specific transition errors highlight key areas for future model refinement.

### 3.2 Spatial focus of the model during key surgical phases

**Figure 5:**
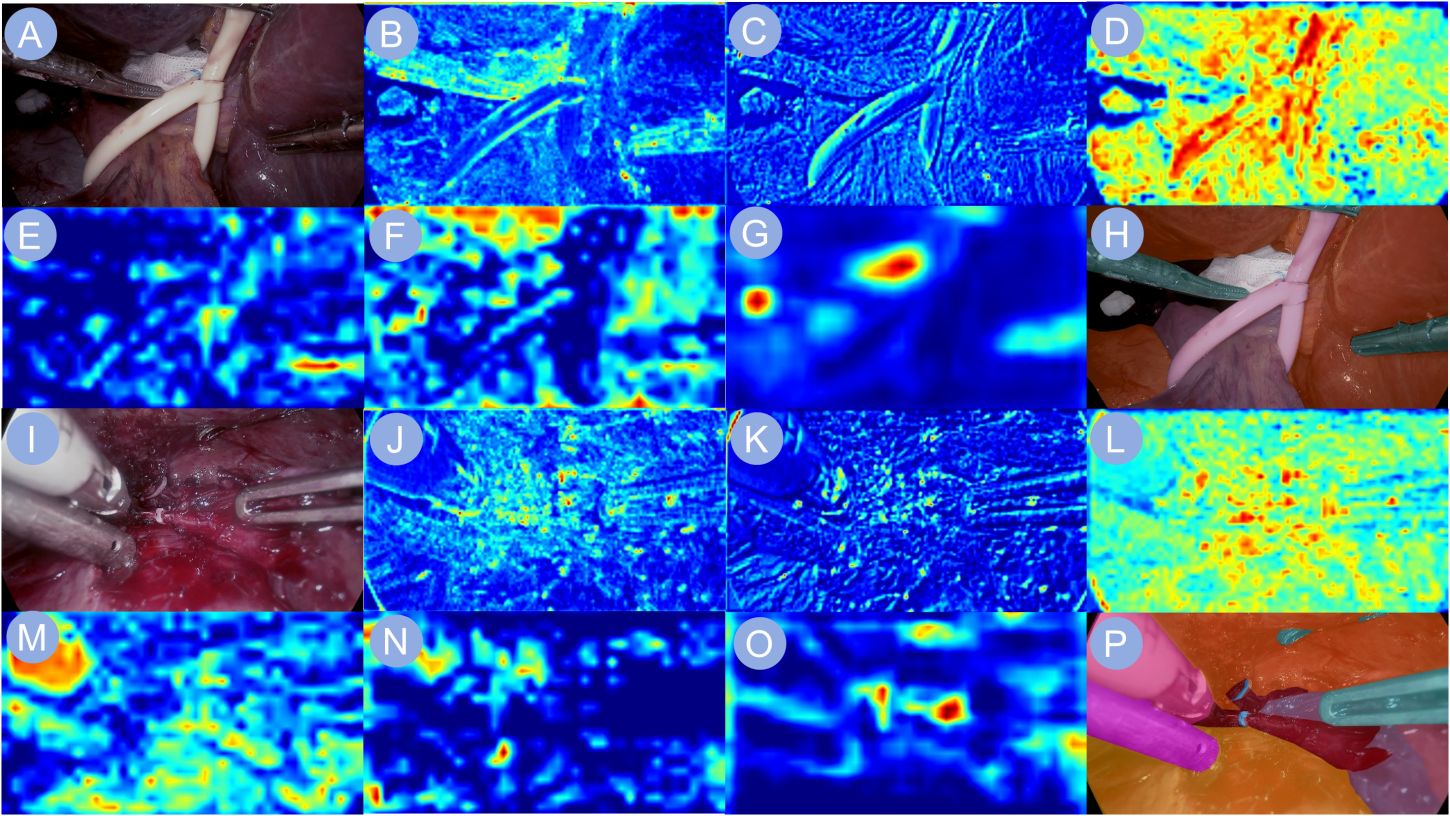
Heatmap visualizations of neural network activations. Heatmap visualizations of neural network activation are shown to illustrate the model’s response during phase recognition in liver surgery. **(A, I)** depict the input surgical images. **(B–G, J–O)** display the corresponding feature maps extracted by the backbone network, capturing key visual cues relevant to the identification of the first hepatic portal occlusion phase and the hepatic pedicle dissection phase, respectively. **(H, P)** present the final output images, highlighting the network’s interpretive focus for accurate surgical phase classification.

To investigate the model’s visual attention during critical operative tasks, we analyzed its multi-level feature extraction across two representative stages: first hepatic hilum occlusion and hepatic pedicle dissection (**Figure 5**). The network gradually constructed semantic representations by extracting local textures and anatomical boundaries from raw laparoscopic images. During the hilum occlusion phase, the activation maps concentrated around the portal vein and the site of vascular clamping, successfully capturing the convergence zone of the hepatic triad. In the pedicle dissection phase, the model’s focus shifted toward the hepatic artery and bile duct trajectories, aligning well with the surgeon’s operative field. The final output heatmaps exhibited high-intensity responses localized precisely over the regions of surgical manipulation, reflecting accurate anatomical comprehension by the network. These attention distributions were tightly aligned with intraoperative targets, suggesting effective feature learning in anatomically complex environments.

### 3.3 Image clarity evaluation

**Table 5** summarizes the precision, recall, F1-score, specificity, and overall accuracy of the ISA in judging the image clarity in each phase. The ISA achieved the highest AUC (0.96) in Phase 1 (**Figure 1D**), indicating its strongest ability to discriminate Phase 1. By contrast, the lowest AUC (0.87) and lowest accuracy (0.85) were observed in Phase 3, indicating its relatively weaker performance in recognizing the procedures in Phase 3.

**Table 5:** Classification performance metrics by phase.

Phase vs All	Precision	Recall	Specificity	F1 Score	Accuracy	AUC	Best threshold
Phase 0	0.57	0.77	0.89	0.66	0.87	0.88	1.76
Phase 1	0.62	0.89	0.89	0.73	0.89	0.96	1.77
Phase 2	0.54	0.83	0.86	0.66	0.86	0.91	1.72
Phase 3	0.53	0.75	0.87	0.62	0.85	0.87	1.71
Phase 4	0.55	0.85	0.86	0.67	0.86	0.92	1.70
Phase 5	0.63	0.87	0.90	0.73	0.89	0.93	1.82

The ISA achieves high recall (typically 0.83–0.89) and accuracy (>85%) in all phases. Precision ranges from ~0.55 to 0.63, and F1 scores are around 0.66–0.73. Notably, AUC values are 0.88–0.96, indicating a strong discriminatory power of the ISA in each phase classification.

### 3.4 Performances of the ISA across multiple cohorts

**Figure 6:**
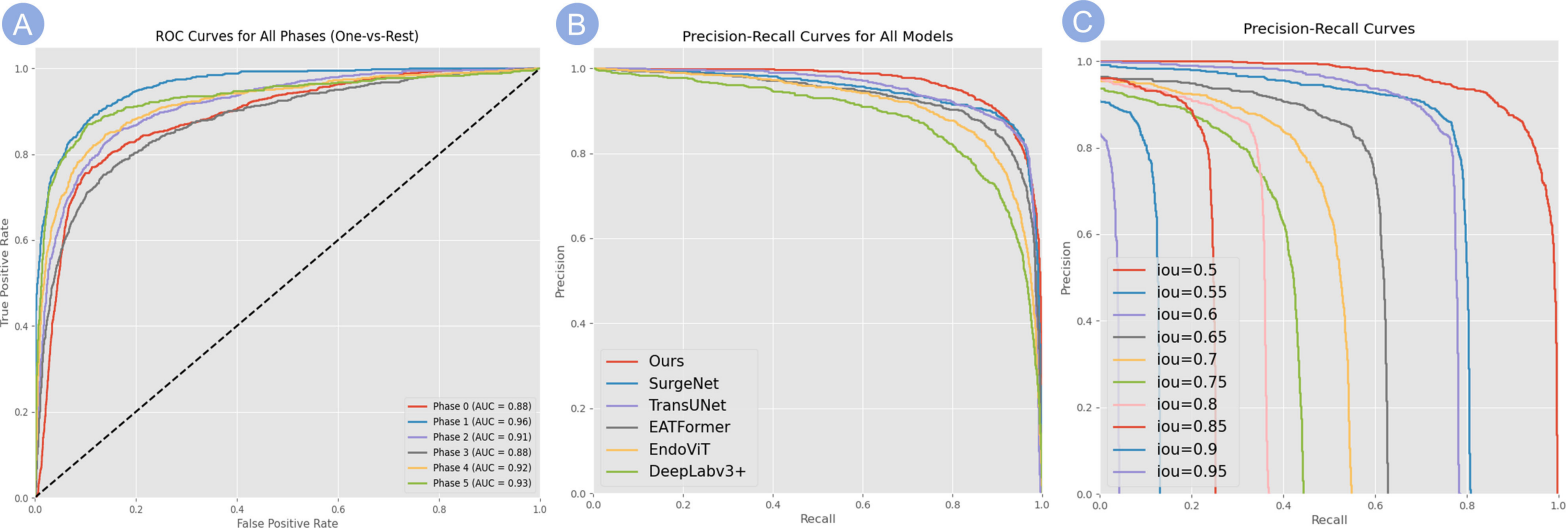
Evaluation of model classification performance. **(A)** ROC curves for the classification of all surgical phases (One-vs-Rest). The high AUC values for each phase demonstrate the model’s robust discriminatory power. **(B)** PR curves comparing our model’s performance against several baseline methods, showing that our model consistently achieves higher precision across varying recall levels. **(C)** PR curves for our model evaluated at increasingly stringent IoU thresholds (from 0.5 to 0.95), demonstrating robust performance even at high IoU values and reflecting its strong spatial localization capabilities.

As shown by the results from the validation cohorts (**Figure 6A**), the ISA achieved the highest AUC (0.9598) in Phase 1, followed by Phase 5 (0.93), Phase 4 (0.92), Phase 2 (0.9137). In Phase 0 and Phase 3, the AUCs were slightly lower (0.8839, 0.8776, respectively), reflecting the relatively weaker yet still reliable discriminatory ability of the ISA.

Notably, the ISA achieved AUC values above 0.87 across all phases of the procedure, indicating a robust and consistent discriminative capability for key frame identification in surgical videos irrespective of stage. Moreover, the performances in recognizing static background and dynamic key phases showed a notable disparity, suggesting that a higher degree of visual complexity (i.e., richer visual information) could enable a more accurate recognition, as shown by that in active surgical scenes, the ISA showed a stronger performance in reading frames. Overall, the consistently high AUC values at each phase demonstrated the model’s stable discriminatory ability.

### 3.5 Performances of the model

As shown in **Table 3**, our method achieves the best performance across most evaluation metrics. Specifically, it obtains the highest values in AP50 (95.2%), AP75 (65.4%), and AP50:95 (62.1%), outperforming the second-best method SurgeNet by 2–4 percentage points. It also ranks first in APL (64.8%), indicating better performance in detecting large anatomical structures. While SurgeNet achieves a slightly higher APM (55.6%), our method remains competitive at 54.3%, demonstrating consistent performance across different object scales.

Regarding efficiency, our model achieves a frame rate of 19.2 FPS, significantly faster than other methods such as TransUNet (15.3 FPS) and DeepLabv3+ (10.1 FPS). This indicates that our method is not only accurate but also practical for real-time applications in clinical scenarios.

The PR performance of our model is detailed in **Figures 6B, C**. **Figure 6B** compares our model to several baselines, demonstrating that our method consistently maintains higher precision across varying recall levels. Furthermore, as shown in **Figure 6C**, our model maintains robust performance even under stricter IoU thresholds (e.g., 0.85 and 0.9), reflecting its strong spatial localization capabilities.

In summary, the results validate the effectiveness and robustness of our method in terms of both detection accuracy and inference speed, highlighting its potential for real-world medical image analysis tasks.

### 3.6 Temporal phase prediction across the full surgical timeline

We further evaluated the model’s temporal prediction performance over the entire course of laparoscopic liver resection, dividing the procedure into five sequential phases and comparing model outputs to expert-annotated ground truth. Without post-processing, the model was able to reproduce the general phase order, though minor misclassifications occurred at transitional boundaries—particularly between the second hepatic portal anatomy and portal vein dissection stages. After applying temporal smoothing and transition constraints, the predicted phase sequence exhibited improved continuity, reduced fragmentation, and better alignment with surgical annotations. In low-motion frames such as post-resection hemostasis observation, the model maintained stable predictions, indicating reliable temporal awareness and rhythmic phase modeling even in visually ambiguous intervals.

### 3.7 Performance on the independent external validation cohort

To rigorously assess the model’s generalizability, we evaluated its performance on a completely independent external validation cohort, which consisted of 122 surgical videos from a center whose data was not used for training. The ISA was applied to this unseen dataset without any retraining or fine-tuning.

On this external cohort, the model demonstrated strong and consistent performance, achieving an average phase recognition accuracy of 89.5%, which is comparable to the 91% accuracy observed in the internal cross-validation. Key performance metrics, including precision, recall, and F1-score, also remained robust, confirming that the model did not overfit to the training data and can generalize effectively to different surgical teams and environments. The detailed performance on the external cohort is summarized in **Table 6**.

**Table 6:** Key performance metrics on the external validation cohort (N=122).

Metric	Average value
Phase Recognition Accuracy	89.5%
Area Under the Curve (AUC)	0.90
Precision	0.88
Recall	0.89
F1-Score	0.88

This table presents the core performance metrics of the ISA model on the independent external validation cohort, which was not used during training, to assess the model's generalizability.

## 4 Discussion

Our development and multi-center validation of the ISA system directly addresses several key challenges recently highlighted in the surgical AI literature. While many studies have focused on single-task excellence, our approach emphasizes a multi-task framework that maintains real-time performance, a critical requirement for clinical adoption (20). Furthermore, by creating a large, multi-center dataset and rigorously validating our model on an independent external cohort, we contribute to solving the issues of data scarcity and model generalizability that are frequently cited as major hurdles in the field (21). Our work therefore represents a significant step toward translating AI from a research concept into a clinically valuable tool, as envisioned by recent reviews (24).

Compared with previous single-task AI systems used in laparoscopic surgery, such as those focused solely on tool tracking or static segmentation, the ISA achieves a comprehensive integration of intraoperative visual information (21). The average classification accuracy exceeded 89% across key phases, with AUCs consistently above 0.87. Notably, our approach outperformed SurgeNet, TransUNet, and EndoViT in both segmentation accuracy (AP50: 95.2%) and frame rate (19.2 FPS), providing not only precision but also practical operability in real surgical environments. These metrics collectively support the reliability of ISA as a real-time clinical decision support tool (22–24).

From an oncological perspective, achieving precise anatomical exposure and reliable intraoperative phase control is essential in liver cancer resection. The ISA’s ability to evaluate phase-specific image clarity and detect critical procedural transitions (e.g., hilum dissection and hemostasis) may directly contribute to complete tumor excision and reduced intraoperative complications. By alerting the surgeon in real time when visual clarity is compromised, the system is designed to mitigate the risks of transecting tissue with inadequate visualization. Whether this function ultimately translates into a reduced incidence of residual tumor warrants investigation in future prospective studies. Although our study did not evaluate long-term oncologic outcomes, the integration of ISA into hepatobiliary workflows may ultimately translate into reduced margin positivity and improved surgical radicality, warranting future investigation.

Despite the promising results, this study has several limitations. The primary limitation is that our validation is confined to technical metrics of accuracy and speed, rather than clinical endpoints. While our system’s ability to accurately identify surgical phases and assess image clarity suggests a strong potential for improving safety, we did not measure its direct impact on outcomes such as operative time, blood loss, or complication rates (24). Therefore, the clinical benefits of the ISA remain a well-founded hypothesis that requires rigorous validation in future prospective, randomized controlled trials. Second, although the dataset is relatively large and multi-institutional, it may not fully capture the heterogeneity of all intraoperative environments, especially in complex tumor resections involving vascular invasion or cirrhotic livers. Third, the current ISA system relies exclusively on endoscopic video input; incorporation of multimodal data such as intraoperative ultrasound or fluorescence imaging may further enhance decision-making accuracy (25, 26).

In conclusion, the proposed ISA demonstrates high accuracy, robustness, and real-time responsiveness in phase-specific analysis during laparoscopic liver surgery. Preliminary feedback from participating surgeons suggests that the system enhances intraoperative decision-making, particularly by clarifying critical transitions such as hemostasis and hepatic hilum dissection. This study exemplifies how AI can bridge the gap between real-time endoscopic imaging and surgical decision-making, supporting procedural consistency and situational awareness.

However, it is important to acknowledge the model’s potential limitations and “failure modes,” particularly in challenging clinical scenarios. As the system relies on visual input, its performance could be compromised by severe intraoperative bleeding that completely obscures the camera, extensive adhesions from reoperations that alter typical anatomy, or rare anatomical variations not well-represented in the training data. Addressing these challenges will be a key direction for future model improvements and is essential for ensuring the system’s reliability in the full spectrum of surgical situations.

Despite these considerations, with continued optimization and integration into clinical workflows, the ISA holds strong potential to improve intraoperative safety and to standardize surgical procedures—particularly in oncologic contexts where precision and margin control are critical. Future prospective trials are warranted to evaluate its clinical impact on operative time, complication rates, and long-term oncologic outcomes. Ultimately, the intelligent vision systems demonstrated by ISA could serve as a foundational component for future integrated platforms that provide intelligent intraoperative navigation and quality control in minimally invasive oncologic surgery.

## Data Availability

The raw data supporting the conclusions of this article will be made available by the authors, without undue reservation.
