# Supplementary figures and images for: Development of an AI-driven digital assistance system for real-time safety evaluation and quality control in laparoscopic liver surgery
Source: Front Oncol. 2025 Oct 8;15:1678525. doi: 10.3389/fonc.2025.1678525 (PMC12541588; doi:10.3389/fonc.2025.1678525)

## Slide 1
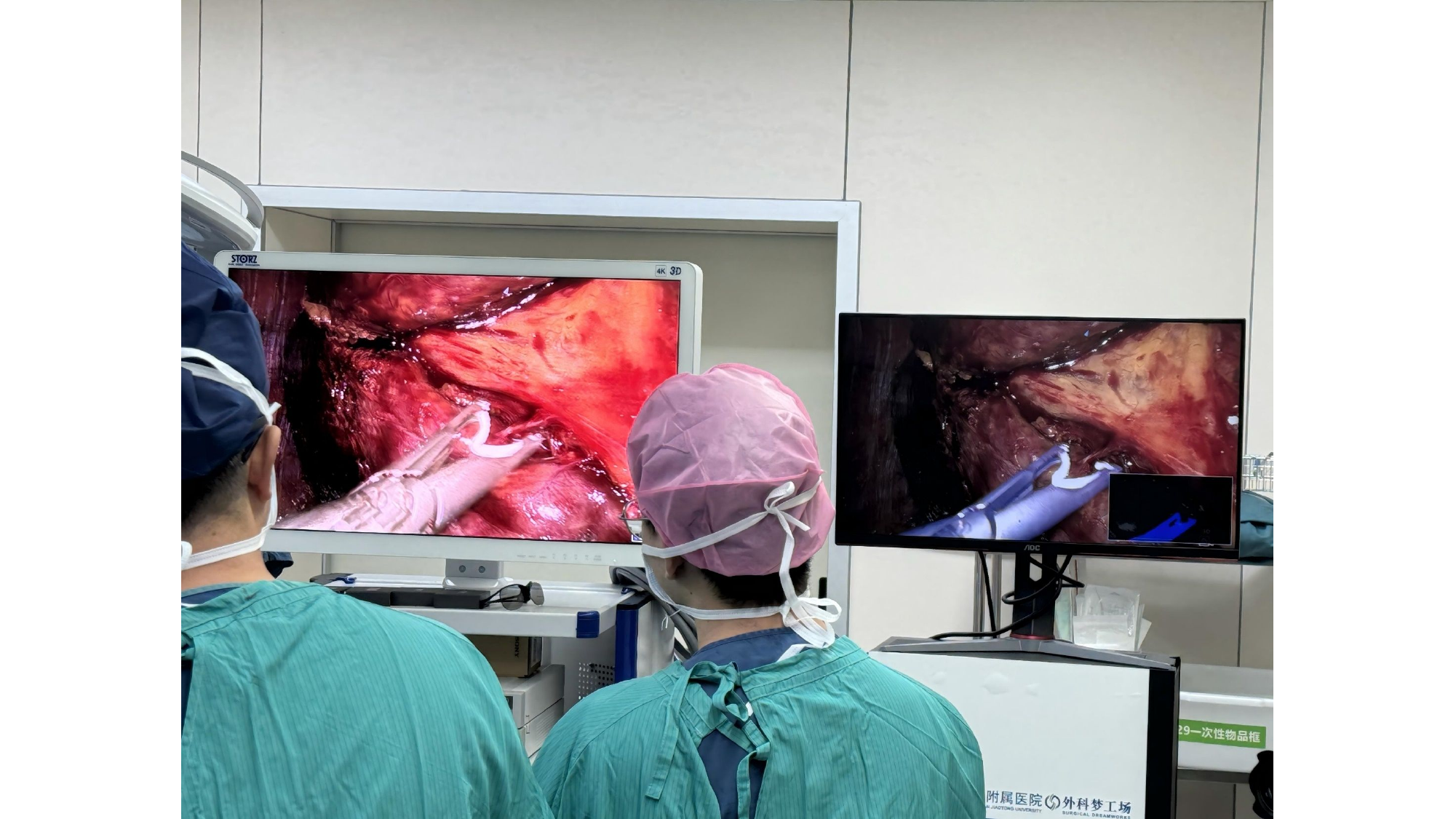

## Slide 2
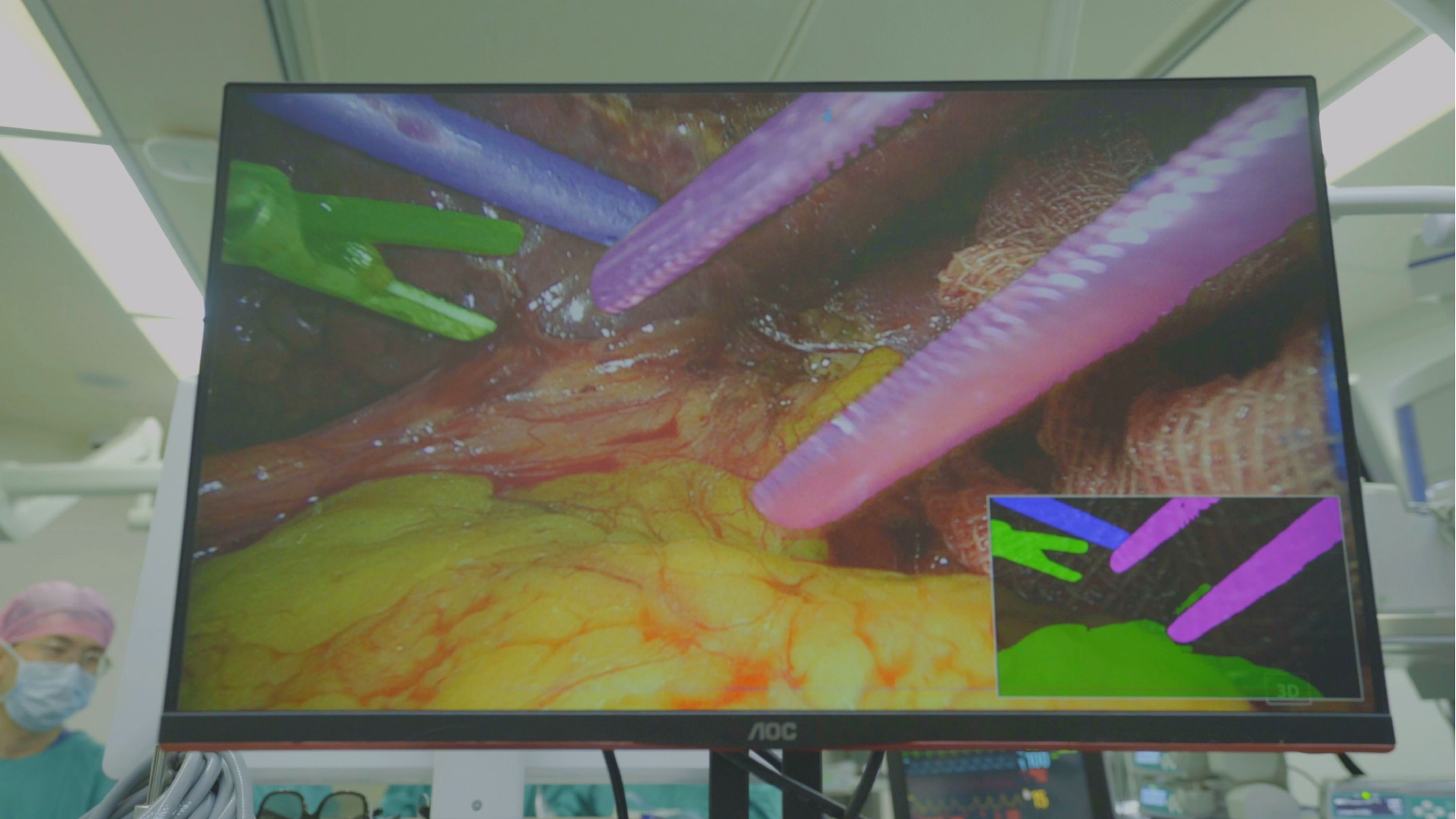

#

Supplement: Supplementary file 1 [file Presentation1.pptx]
